# Predição da Propensão à Cardiopatia Atrial e Fibrilação Atrial Paroxística em Pacientes com Acidente Vascular Cerebral Embólico de Origem Indeterminada (ESUS)

**DOI:** 10.36660/abc.20240213

**Published:** 2025-01-08

**Authors:** Ahmed Kishk, Mohamed E. Abdeldayem, Mohamed A. Khalil, Mohammed Elbarbary

**Affiliations:** 1 Cardiovascular Medicine Department Faculty of Medicine Tanta University Tanta Egito Cardiovascular Medicine Department, Faculty of Medicine, Tanta University, Tanta – Egito; 2 Neuropsychiatry Department Faculty of Medicine Tanta University Tanta Egito Neuropsychiatry Department, Faculty of Medicine, Tanta University, Tanta – Egito

**Keywords:** Fibrilação Atrial, Cardiopatia Atrial, Acidente Vascular Cerebral

## Abstract

**Fundamento:**

Ainda há uma população significativa de pacientes com acidente vascular cerebral embólico de origem indeterminada (ESUS) cuja causa específica atribuível ao acidente vascular cerebral permanece desconhecida.

**Objetivos:**

Nossa pesquisa teve como objetivo avaliar parâmetros clínicos, eletrocardiográficos, laboratoriais e ecocardiográficos que podem predizer a propensão à fibrilação atrial paroxística (FAP).

**Métodos:**

Inscrevemos setenta e cinco pacientes ESUS que estavam em ritmo sinusal no momento do diagnóstico de acidente vascular cerebral para serem submetidos a monitoramento Holter de 7 dias no hospital, teste para Pro-BNP e um exame ecocardiográfico padrão. Para análise estatística, um valor de P < 0,05 foi considerado significativo.

**Resultados:**

A idade média dos 75 pacientes ESUS foi de 58 anos. 60% dos pacientes eram do sexo masculino, e a condição concomitante mais prevalente foi hipertensão (53,3%). Quarenta pacientes tinham cardiopatia atrial, e 15 pacientes tiveram episódios de FAP. Hipertensão e E/e- > 12 foram preditores independentes de cardiopatia atrial, com valores de p de 0,001 e 0,02, respectivamente. Em pacientes com cardiopatia atrial, foi realizada análise de regressão multivariável; velocidade de força terminal da onda P na derivação V (PTFV) > 5000 mV.ms, índice de volume do átrio esquerdo > 34 ml/m^2^ e fração de ejeção < 45% foram preditores independentes significativos de FA com valores de p significativos de 0,001, < 0,001 e 0,001, respectivamente.

**Conclusões:**

Em pacientes ESUS, a cardiopatia atrial foi prevalente. Hipertensão e uma razão E/e- maior que 12 foram preditores independentes para ela. A análise de regressão multivariável identificou PTFV1 > 5000 mV.ms, índice de volume de AE > 34 ml/m^2^ e fração de ejeção < 45% como preditores independentes para fibrilação atrial de início recente.

## Graphical abstract

**Figura Central:**
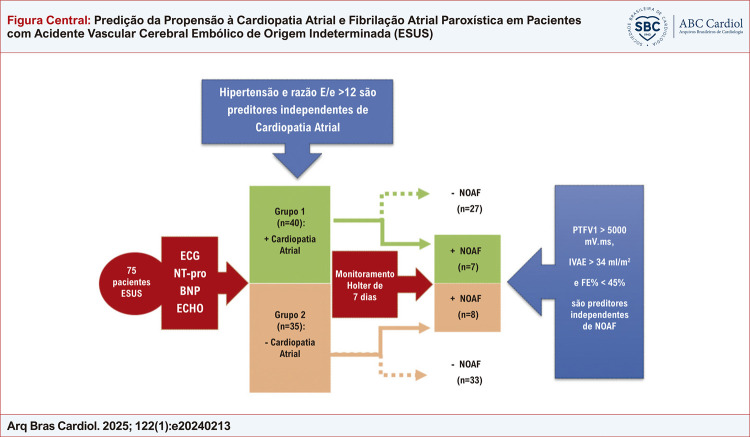
: Predição da Propensão à Cardiopatia Atrial e Fibrilação Atrial Paroxística em Pacientescom Acidente Vascular Cerebral Embólico de Origem Indeterminada

ECG: eletrocardiograma; ECHO: ecocardiograma; NT-pro BNP: peptídeo pronatriurético N-terminal; +: com; -: sem; NOAF: fibrilação de início recente; PTFV1: força terminal da onda P em V1; E/e-: razão de E para E’ onde a velocidade de pico do fluxo transmitral diastólico inicial e e- é a velocidade de pico do movimento anular mitral diastólico inicial conforme determinado pelo Doppler de onda pulsada; IVAE: índice de volume do átrio esquerdo; FE%: fração de ejeção.

## Introdução

A fibrilação atrial (FA) é uma doença associada a um risco elevado de acidente vascular cerebral isquêmico, insuficiência cardíaca, declínio cognitivo, demência, infarto do miocárdio, morte súbita cardíaca e morte por todas as causas.^1,2^

O tromboembolismo pode ocorrer em casos de remodelação do átrio esquerdo (AE) ou cardiopatia atrial, que é caracterizada por alterações estruturais e funcionais, como fibrose atrial, disfunção endotelial e inflamação. Isso significa que a presença de FA em um eletrocardiograma (ECG) nem sempre é necessária para que o tromboembolismo se manifeste.^3^

Há também evidências de dissociação eletromecânica atrial em pacientes com amiloidose cardíaca que podem estar em ritmo sinusal eletrocardiográfico, apesar dos átrios fibrilantes.^4^ Além disso, biomarcadores cardíacos indicativos de disfunção atrial estão associados a um risco aumentado de acidente vascular cerebral e sua recorrência, mesmo na ausência de FA.^5^

A cardiopatia atrial pode ser definida por pelo menos um dos seguintes critérios: nível sérico de pró-peptídeo natriurético cerebral N-terminal (NT-pro BNP) maior que 250 Pg./ml; velocidade de força terminal da onda P na derivação V1 (PTFV1) no ECG maior que 5000 µv.ms; ou aumento atrial esquerdo (AAE) grave no ecocardiograma (índice de tamanho do AE 3≥ cm/m^2^).^6^

A fibrilação atrial paroxística (FAP) constitui cerca de 25%–62% de todos os casos de FA. Cerca de um terço dos pacientes que apresentaram um acidente vascular cerebral embólico e estavam em ritmo sinusal no momento do diagnóstico provaram ter episódios de FAP quando monitorados com um registrador de loop implantado (ILR).^7^Supõe-se que uma parte de acidente vascular cerebral embólico de origem indeterminada em pacientes (ESUS) com FAPs podem ser subdiagnosticados e submetidos a modalidades de tratamento menos eficazes: aspirina em vez de anticoagulação oral (ACO).^8^

Em nosso trabalho, objetivamos investigar a possibilidade de combinar variáveis eletrocardiográficas, ecocardiográficas e laboratoriais para prever a probabilidade de desenvolvimento de FA paroxística, especialmente em pacientes com ESUS. Esses preditores podem ajudar a gerenciar a população ESUS em vez do caro ILR, especialmente em países de renda média-baixa (LMICs).

Além disso, avaliamos a prevalência de cardiopatia atrial em pacientes ESUS, o que pode contribuir para tromboembolismo mesmo na ausência de FA.

## Métodos

Este estudo prospectivo observacional unicêntrico foi conduzido de junho de 2021 a janeiro de 2023. Incluímos pacientes com 18 anos ou mais que foram diagnosticados com acidente vascular cerebral isquêmico e internados na unidade de tratamento de acidente vascular cerebral, Tanta University Hospital, Egypt.

O ESUS foi diagnosticado de acordo com os critérios de consenso definidos pelo *Cryptogenic Stroke/ESUS International Working Group* como a presença de acidente vascular cerebral isquêmico não lacunar, a ausência de aterosclerose causando estenose luminal ≥50%, fração de ejeção do ventrículo esquerdo (FEVE) ≥30% e nenhuma fonte cardioembólica identificável de embolia.^9^

Pacientes com marcapasso permanente, desfibrilador cardíaco implantado, cirurgia cardíaca recente nos 30 dias anteriores à inscrição e pacientes com diagnóstico de FA em um ECG de repouso feito no momento da inscrição ou nas primeiras 24 horas de monitoramento Holter ou com diagnóstico prévio de FA permanente ou FA persistente foram excluídos.

Todos os pacientes passaram por neuroimagem e investigações vasculares após uma avaliação clínica completa e histórico detalhado. Amostras de sangue foram coletadas para conduzir testes laboratoriais de rotina. O exame cardiológico padrão incluiu ECG em repouso, sete dias de monitoramento cardíaco Holter em regime de internação e ecocardiografia transtorácica 2D.

Cada paciente recebeu um ECG de superfície padrão de 12 derivações, registrado a uma velocidade de 25 mm/s e uma voltagem de 10 mm/mV. Um único observador que era cego para o grupo de pacientes analisou os ECGs em zoom digital quatro vezes.

Os seguintes índices da onda P no ECG foram definidos e avaliados da seguinte forma:

- O PTFV1 foi calculado como o produto da amplitude e da duração da deflexão terminal descendente da onda P na derivação V1.^10^ O PTFV1 foi medido somente se a morfologia da onda P na derivação V1 tivesse um componente negativo ou bifásico e foi excluído quando havia uma onda P positiva. As medições foram feitas no ECG de papel de admissão usando paquímetros digitais em mm e foram então convertidas para μV e ms usando a calibração de ECG de 10 mm/mV e 25 mm/s.
- As durações da onda P foram medidas nas derivações V1 (PWDV1) e II (PWDII). Foi definido como o tempo do início mais precoce da atividade da onda P nas derivações até a atividade mais recente da onda P nessas derivações.^10^
- A dispersão da onda P é definida como a diferença entre as durações máxima e mínima da onda P medidas em todas as derivações derivadas do ECG.^11^

Um monitoramento Holter hospitalar de 7 dias foi aplicado a todos os pacientes incluídos para medir a prevalência de fibrilação atrial de início recente (NOAF). NOAF foi definida como evidência de ECG (ou Holter) de ritmo sustentado irregular sem evidência de atividade atrial discreta em pacientes com histórico negativo de FA persistente ou permanente.^12^

Cada paciente foi submetido a ecocardiografia 2D utilizando uma máquina GE Vivid S5 com transdutores phased-array (M5S) (General Electric, Horten, Noruega) durante a mesma internação hospitalar. De acordo com as recomendações da Sociedade Americana de Ecocardiografia, medidas padrão de vários parâmetros ecocardiográficos e Doppler foram tomadas com a determinação da FEVE usando o método biplano de Simpson, índice de volume do átrio esquerdo (IVAE) calculado a partir de vistas apicais de quatro e duas câmaras e razão E/e^-^

Os pacientes foram divididos em dois grupos de acordo com a presença ou ausência de cardiopatia atrial: Grupo I, composto por indivíduos com diagnóstico de cardiopatia atrial, e Grupo II, composto por aqueles sem cardiopatia atrial.

A cardiopatia atrial foi definida por pelo menos um dos seguintes critérios: NT-pro BNP maior que 250 Pg./ml; PTFV1 no ECG maior que 5000 µv.ms; ou AAE grave no ecocardiograma (índice de tamanho do AE 3≥ cm/m^2^).^6^

Posteriormente, os pacientes foram divididos em dois subgrupos com base na ocorrência de FA de início recente: subgrupo A, que teve pelo menos um episódio de FA de início recente com duração superior a 30 segundos, e subgrupo B, que não teve episódios de FA.

### Análise estatística

- As variáveis contínuas apresentaram distribuição normal (verificada pelo teste de Kolmogorov-Smirnov), sendo descritas por média ± desvio padrão e testadas pelo teste t de Student não pareado.
- As variáveis categóricas foram apresentadas como frequências absolutas e relativas e analisadas usando o teste χ^2^ ou o teste exato de Fisher, conforme apropriado.
- Foi empregado um modelo de regressão logística multivariável ajustando para idade, sexo, hipertensão e diabetes mellitus, e os resultados foram apresentados.
- A análise estatística foi feita online usando DATAtab: Calculadora de Estatística Online,^14^ onde um valor de p < 0,05 foi considerado estatisticamente significativo.

## Resultados

A identificação inicial de 82 pacientes consecutivos com ESUS resultou na exclusão de 7 pacientes devido a problemas técnicos na obtenção de visualizações ecocardiográficas suficientes (dois casos), transferências para outros hospitais antes de completar o estudo Holter por sete dias (três casos) ou mortes hospitalares (dois casos). Assim, a coorte do estudo foi criada pela inclusão dos 75 pacientes finais.

Os pacientes incluídos foram então divididos em dois grupos com base na presença ou não de cardiopatia atrial. O Grupo I representou a população com cardiopatia atrial e incluiu 40 pacientes, e o Grupo II representou a população sem cardiopatia atrial e incluiu 35 pacientes. Posteriormente, cada grupo foi subdividido em dois subgrupos, A e B, com base na presença ou não de FA, respectivamente.

Novos episódios de FA hospitalar foram registrados em 15 pacientes (representando 20% do total de 75 ESUS), 7 pacientes no grupo I (17,5%) e 8 pacientes no grupo II (22,8%).

A idade média dos pacientes incluídos foi de 58 anos. Pacientes com cardiopatia atrial eram significativamente mais velhos do que aqueles sem cardiopatia atrial. A maioria dos pacientes era do sexo masculino. Hipertensão foi a comorbidade mais comum. Pessoas com diabetes tinham mais probabilidade de estar no grupo II. Não houve diferença estatisticamente significativa entre os dois grupos considerando gênero, hipertensão, doença arterial coronária, acidente vascular cerebral/AIT anterior ou múltiplos territórios vasculares de infarto (Tabela 1).

**Tabela 1:** – Características clínicas basais da população incluída nos dois grupos

Variável	Grupo I: Com cardiopatia atrial (n= 40)	Grupo II: Sem cardiopatia atrial (n= 35)	Valor p*
Idade	60,5±6,39	55,14±3,57	<0,01
Gênero masculino	25 (62,5%)	20 (57,1%)	0,637
Tabagismo	7 (17,1%)	8 (23,4%)	0,072
IMC (kg/m^2^)	27,6 ± 2,9	26,6 ± 3,1	0,126
Diabetes	6 (8%)	14 (18,67%)	0,015
Hiperlipidemia	17 (22,67%)	13 (17,33%)	0,637
Doença cardíaca isquêmica	33 (44%)	27 (36%)	0,563
Insuficiência cardíaca	5 (11,9%)	5 (14,2%)	0,82
AIT/AVC anterior	7 (9,33%)	8 (10,67%)	0,563
Infrações em múltiplos territórios vasculares	7 (9,33%)	13 (7,33)	0,55

*Foi realizado um teste Chi^2^. Valor de p < 0,05 foi considerado estatisticamente significativo. IMC: índice de massa corporal; AVC: acidente vascular cerebral

Em relação às informações ecocardiográficas, não houve diferenças estatisticamente significativas ao comparar diâmetro do AE, índice de diâmetro do AE, índice de volume do AE, volume do AE, índice de volume diastólico final do VE e índice de massa ventricular esquerda (IMVE), (Tabela 2).

**Tabela 2:** – Achados laboratoriais, dados ecocardiográficos e características do ECG em ambos os grupos

Variável	Grupo I: Com Cardiopatia Atrial (N= 40)	Grupo II: Sem Cardiopatia Atrial (N= 35)		Valor p
Resultados laboratoriais				
TFGe (média ± desvio padrão)	82,43 ± 6,69	78,94±10,32		0,093
HBA1C	6,48±0,66	6,91±1,26		0,081
Colesterol LDL	104,35±24,42	100,74±34,15		0,605
Achados ecocardiográficos				
Diâmetro do AE, mm	36,59 ±4,4	35,54±3,41		0,255
Índice de diâmetro do AE, mm/m^2^	21,45±2,53	20,85±1,99		0,265
Volume de AE, ml	53,68±10,38	53,66±9,09		0,994
IVAE, ml/m^2^	31,7±6,36	31,41±5,13		0,827
IVDFVE, mL/m^2^	58,5±2,72	59,13±3,42		0,375
IMVE, g/m^2^	98,4±2,99	98,03±4,53		0,677
**Índices da onda P no ECG**				
PTFV1, mV.ms	36,88±3,8	34,7±3,94		0,017
Duração da onda P, ms	113,7±0,83	113,43±0,94		0,182
Dispersão da onda P	39,85±0,22	39,74±0,49		0,243

TFGe: taxa de filtração glomerular estimada; HBA1C: hemoglobina glicada; LDL: lipoproteínas de baixa densidade; AE: átrio esquerdo; IVDFVE: índice de volume diastólico final do VE; IVAE: índice de volume do átrio esquerdo; IMVE: índice de massa ventricular esquerda; PTFV1: força terminal da onda P em V1.

PTFV1 foi estatisticamente significativamente maior no Grupo I do que no Grupo II. No entanto, a diferença entre os dois grupos quanto à duração da onda P e dispersão da onda P não foi estatisticamente significativa.

Análise de regressão multivariável foi realizada para determinar preditores independentes de cardiopatia atrial em pacientes ESUS. Os resultados mostraram que a hipertensão e a razão E/e^-^ foram estatisticamente significativas (Tabela 3).

**Tabela 3:** – Análise de regressão multivariável mostrando preditores independentes de cardiopatia atrial em pacientes ESUS

Variável	Valor p	IC 95%
Idade	0,355	0,58 - 4,55
Tabagismo	0,467	0,28 - 1,8
Hipertensão	0,001	2,01 - 17,88
HBA1C > 6,5%	0,997	0 - Infinito
Dislipidemia	0,068	0,94 - 6,21
IM Prévio	0,398	0,43 - 8,17
E/e-	0,02	1,2 – 8,19
IMVE, ml/m^2^	0,049	1 - 1,36
FE < 45%	0,998	0,57 - 1,5
Duração da onda P (ms)	0,944	0,61 - 1,7
Dispersão da onda P (ms)	0,799	0,32 - 4,31

HBA1C: hemoglobina glicada; IM: infarto do miocárdio; AE: átrio esquerdo; IVAE: índice de volume do átrio esquerdo; E/e-: razão de E para e-, onde E é a velocidade de pico do fluxo transmitral diastólico inicial e e- é a velocidade de pico do movimento anular mitral diastólico inicial; FE: fração de ejeção; PTFV1: força terminal da onda P em V1.

**Figura 1:**
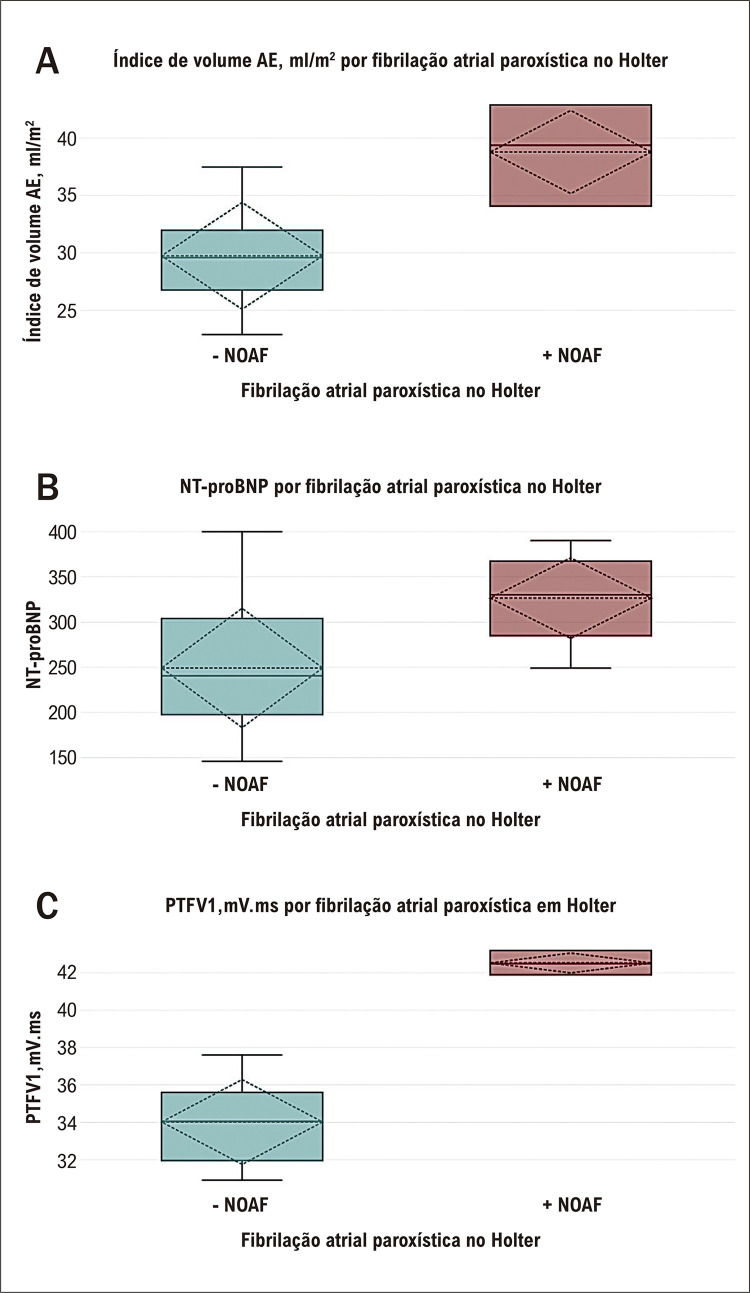
– Box blot comparando A) índice de volume de AE, ml/m2, B) NT-proBNP e C) PTFV1, mV.ms entre pacientes com NOAF (+NOAF) e pacientes sem NOAF (- NOAF).

Em relação aos episódios de FA, NOAF foi registrado usando sete dias de monitoramento Holter em 15 pacientes. O grupo NOAF mostrou um índice de volume de AE significativamente maior, um NT-pro BNP aumentado e um PTFV1 maior em comparação aos pacientes sem NOAF (Figura 1, Tabela 4).

**Tabela 4:** – Comparação dos determinantes da cardiopatia atrial (índice de volume do AE, NT-proBNP e PTFV1) entre pacientes com NOAF e pacientes sem NOAF

Variável	NOAF durante 7 dias de monitoramento Holter n= 15	Não NOAF durante 7 dias de monitoramento Holter n= 60	Valor p*
Índice de volume do AE, ml/m^2^ (M ± DP)	38,8 ± 3,74	29,76 ± 4,69	<0,001
NT-proBNP pg/ml (M ± DP)	249,27 ± 66,52	326,53 ± 46,05	<0,001
PTFV1,mV.ms (M ± DP)	42,53 ± 0,55	34,02 ± 2,29	<0,001

NOAF: fibrilação atrial de início recente; NT-proBNP: peptídeo natriurético pró-atrial N-terminal; PTFV1: força terminal da onda P em V1. * Foi utilizado o teste-T. Valor de p < 0,05 foi considerado significativo.

Para identificar os determinantes independentes da FA em pacientes ESUS com cardiopatia atrial, foi realizada uma análise de regressão multivariável. Descobrimos que PTFV1 > 5000 mV.ms, índice de volume do AE > 34 ml/m^2^ e fração de ejeção < 45% foram preditores independentes significativos para FA (Tabela 5).

**Tabela 5:** – Análise de regressão multivariável mostrando preditores independentes de fibrilação atrial em pacientes ESUS com cardiopatia atrial

	Valor p	IC 95%
Idade > 65	0,175	0,54 - 25,93
Tabagismo	0,4	0,07 - 3,01
Hipertensão	0,186	0,45 - 4,34
HBA1C > 6,5%	0,877	0,08 - 8,77
Dislipidemia	0,173	0,43 - 41,8
Infarto do miocárdio prévio	0,137	0,65 - 40,95
NT-pro BNP >250 pg/ml	0,087	0,61 - 59,45
E/e- >12	0,038	0 - Infinito
Índice de tamanho de AE 3 ≥ cm/m^2^	0,062	0 - Infinito
IVAE > 34 ml/m^2^	<0,001	0 - Infinito
FE < 45%	0,001	0,43 - 0,99
PTFV1 > 5000 Μv.ms	0,001	1 - 1,02
Duração da onda P (ms)	0,014	0,11 - 0,83
Dispersão da onda P (ms)	0,433	0,11 - 121,32

HBA1C: Hemoglobina glicada; NT-proBNP: peptídeo natriurético pró-atrial N-terminal: E/e-: razão de E para E’ onde Velocidade de pico do fluxo transmitral diastólico inicial e e- é Velocidade de pico do movimento anular mitral diastólico inicial conforme determinado pelo Doppler de onda pulsada; AE: átrio esquerdo: IVAE: índice de volume do átrio esquerdo; FE: fração de ejeção; PTFV1: Força terminal da onda P em V1.

## Discussão

Em 2014, o conceito de acidente vascular cerebral embólico de fonte indeterminada (ESUS) foi desenvolvido e publicado. Aproximadamente 20% de todos os acidentes vasculares cerebrais isquêmicos são causados por acidente vascular cerebral embólico de fonte indeterminada (ESUS), um subtipo de acidente vascular cerebral isquêmico criptogênico.^15,16^

A FA está significativamente correlacionada com acidentes vasculares cerebrais isquêmicos, contribuindo para a morbidade e mortalidade globais.^1^ O objetivo deste estudo foi encontrar os potenciais preditores de FA paroxística em pacientes que estavam em ritmo sinusal no momento do diagnóstico de acidente vascular cerebral (AVC). Isso ajudará na formulação de uma estratégia preventiva visando mitigar a ocorrência de AVC embólicos.

Episódios de FA de novo início (NOAF) foram registrados em 20% do total de 75 pacientes ESUS. Essa taxa de identificação de NOAF é semelhante à encontrada por Vera et al.,^17^ que detectaram FA em 24% dos pacientes submetidos a 15 dias de monitoramento Holter-ECG vestível. Além disso, essa ocorrência tem semelhança com o estudo conduzido por Carrazco et al.,^18^ que descobriram que 31% de 101 pacientes com acidente vascular cerebral criptogênico apresentaram FA enquanto eram monitorados por ILR.

Em outros estudos, a taxa de identificação de NOAF usando monitoramento Holter hospitalar por 24 horas, 48 horas e 7 dias é de 4,6%, 6,4% e 12,5%, respectivamente, enquanto a taxa de detecção usando um gravador de loop implantável por 6 meses é de 9%.^19,20^

Em nosso estudo, 53,3% dos pacientes ESUS tinham cardiopatia atrial. Essa proporção é comparativamente maior do que os achados relatados por Ning et al.^21^ e Jalini et al.,^22^ que relataram taxas de prevalência de 42,8% e 26,6%, respectivamente. No entanto, os critérios diagnósticos para cardiopatia atrial propostos por Shirin Jalini et al. foram limitados à presença de uma força terminal da onda p em V1 maior que 5.000 µV.ms ou AAE grave. Notavelmente, esses critérios não incorporaram a inclusão de altos níveis de NT-proBNP acima de 250 pg/ml como um critério diagnóstico.

Em relação ao PTFV1, nosso resultado corresponde ao de Hancock et al.,^23^que descobriram que o PTFV1 anormalmente aumentado é um biomarcador de disfunção atrial esquerda e está associado a alterações fisiopatológicas, como hipertrofia e pressões de enchimento elevadas. Evidências de coortes observacionais sugerem que o PTFV1 elevado está relacionado ao risco de acidente vascular cerebral isquêmico.^24^

Em relação ao IMVE grande > 34 ml/m^2^, a associação pode ser explicada pelo fato de que o aumento do AE carrega um potencial trombogênico provavelmente promovendo estase, lesão endotelial e formação de trombo. Isso está de acordo com Jordan et al.,^25^ que revelaram que o VAE indexado à área de superfície corporal do sujeito está independentemente associado ao desenvolvimento de FA em pacientes ESUS. Além disso, nossos resultados concordam com Yaghi et al.,^6^ que explicou a associação entre aumento do AE e subtipo de acidente vascular cerebral embólico recorrente.

O NT-proBNP elevado é uma medida de disfunção cardíaca, estiramento cardíaco e sobrecarga de volume e é um preditor de FA incidente.^21^ Em nosso estudo, NT-pro BNP elevado > 250 pg/mL foi encontrado em 35 pacientes; deles, apenas 7 pacientes tiveram episódios de FA durante seu monitoramento. Esses resultados correspondem aos de Chen et al.,^26^ que encontraram uma associação entre NT-proBNP elevado e acidente vascular cerebral isquêmico, particularmente do subtipo embólico.

Em contraste com Handke et al.,^27^ que mostraram que a FA é provavelmente mais prevalente entre pacientes mais velhos (>60 anos) com ESUS, não identificamos a idade como um preditor para FA. Isso pode ser atribuído ao nosso pequeno tamanho de amostra, o que limitou nossos resultados.

Um grupo de pacientes ESUS pode se beneficiar de OAC, como demonstrado por Diener et al.,^15^ particularmente aqueles com cardiopatia atrial. Além disso, Merkler et al.^28^ descobriram que a rivaroxabana foi superior à aspirina na redução do risco de acidente vascular cerebral recorrente ou embolia sistêmica entre os participantes do NAVIGATE ESUS com disfunção do VE. As diretrizes canadenses recomendam que pacientes mais velhos com ESUS não lacunar e suspeita de FA sejam submetidos a uma triagem extensiva de FA para identificar pacientes que se beneficiariam de ACO se um diagnóstico claro for encontrado.^12^

Enquanto os estudos RE-SPECT ESUS^29^ e NAVIGATE ESUS^30^ levantaram preocupações sobre a segurança da anticoagulação em pacientes com ESUS, lembramos da importância de avaliar o risco individual de sangramento usando a pontuação HAS-BLED.^31^ A função renal e o hemograma completo devem ser medidos antes de iniciar o ACO e em intervalos regulares depois disso, enquanto o ACO prescrito for dosado adequadamente.

### Limitação do estudo

1. O estudo conduzido foi uma investigação de centro único.
2. O estudo envolveu um número relativamente limitado de casos.
3. Um único observador conduziu a análise do ECG.

## Conclusão

Em pacientes ESUS, a cardiopatia atrial foi prevalente. Hipertensão e uma razão E/e^-^ maior que 12 foram preditores independentes para isso. A análise de regressão multivariável identificou PTFV1 > 5000 mV.ms, índice de volume do AE > 34 ml/m^2^ e fração de ejeção < 45% como preditores independentes para NOAF.
