# Supplementary material for: Predição da Propensão à Cardiopatia Atrial e Fibrilação Atrial Paroxística em Pacientes com Acidente Vascular Cerebral Embólico de Origem Indeterminada (ESUS)
Source: Arq Bras Cardiol. 2025 Jan 8;122(1):e20240213. [Article in Portuguese] doi: 10.36660/abc.20240213 (PMC11809893; doi:10.36660/abc.20240213)
Supplement: Translation

# Translation: Predicting the Propensity of Atrial Cardiopathy and Paroxysmal Atrial Fibrillation in Patients with Embolic Stroke of Undetermined Source (ESUS)

**DOI:** 10.36660/abc.20240213i

**Authors:** Mohammed Elbarbary, Ahmed Kishk, Mohamed E. Abdeldayem, Mohamed A. Khalil

**Affiliations:** 1 Egypt Cardiovascular Medicine Department, Faculty of Medicine, Tanta University, Tanta – Egypt; 2 Egypt Neuropsychiatry Department, Faculty of Medicine, Tanta University, Tanta – Egypt

**Keywords:** Atrial Fibrillation, Atrial Cardiopathy, Stroke

## Abstract

**Background:**

There is still a significant population of patients with embolic stroke of Undetermined Source (ESUS) whose specific attributable cause of the stroke remains unknown.

**Objectives:**

Our research aimed to assess clinical, electrocardiogram, laboratory, and echocardiographic parameters that may predict the propensity of paroxysmal atrial fibrillation (PAF).

**Methods:**

We enrolled seventy-five ESUS patients who were in sinus rhythm at the time of stroke diagnosis to undergo in-hospital 7-day Holter monitoring, testing for Pro-BNP, and a standard echocardiographic examination. For statistical analysis, a P-value < 0.05 was considered significant.

**Results:**

The average age of the 75 ESUS patients was 58 years old. 60% of the patients were male, and the most prevalent concomitant condition was hypertension (53.3%). Forty patients had atrial cardiopathy, and 15 patients had PAF episodes. Hypertension and the E/e^-^ > 12 were independent predictors of atrial cardiopathy, with p-values of 0.001 and 0.02, respectively. In patients with atrial cardiopathy, multivariable regression analysis was performed; PTFV > 5000 Mv.ms, LA volume index > 34 ml/m^2^, and ejection fraction < 45% were significant independent predictors of AF with significant p values of 0.001, < 0.001, and 0.001 respectively.

**Conclusions:**

In ESUS patients, atrial cardiopathy was prevalent. Hypertension and an E/e^-^ ratio greater than 12 were independent predictors for it. Multivariable regression analysis identified PTFV1 > 5000 mV.ms, LA volume index > 34 ml/m^2^, and ejection fraction < 45% as independent predictors for new-onset atrial fibrillation.

## Introduction

Atrial fibrillation (AF) is a disease linked to an elevated risk of ischemic stroke, heart failure, cognitive decline, dementia, myocardial infarction, sudden cardiac death (SCD), and all-cause death.^1,2^

Thromboembolism can occur in cases of left atrial (LA) remodeling or atrial cardiopathy, which is characterized by structural and functional changes such as atrial fibrosis, endothelial dysfunction, and inflammation. This means that the presence of AF in an electrocardiogram (ECG) is not always necessary for thromboembolism to manifest.^3^

There is also evidence of atrial electromechanical dissociation in patients with cardiac amyloidosis who may be in electrocardiographic sinus rhythm despite fibrillating atria.^4^ Furthermore, cardiac biomarkers indicative of atrial dysfunction are linked to an increased risk of stroke and its recurrence, even in the absence of AF.^5^

Atrial cardiopathy could be defined by at least one of the following criteria: serum N-terminal pro-brain natriuretic peptide (NT-pro BNP) level greater than 250 Pg./ml; P-wave terminal force velocity in lead V1 (PTFV1) on ECG greater than 5000 µv.ms; or severe left atrial enlargement (LAE) on echocardiogram (LA size index 3≥ cm/m^2^).^6^

Paroxysmal atrial fibrillation (PAF) constitutes about 25%–62% of the whole AF cases. About one-third of patients who presented with an embolic stroke and were in sinus rhythm at the time of diagnosis proved to have PAF episodes when monitored with an implanted loop recorder (ILR).^7^It is hypothesized that a portion of embolic stroke of Undetermined Source patients (ESUS) with PAFs may be underdiagnosed and subjected to less effective treatment modalities: aspirin instead of oral anticoagulation (OAC).^8^

In our work, we aimed to investigate the possibility of combining Electrocardiographic, echocardiographic, and laboratory variables to predict the likelihood of paroxysmal AF development, especially in patients with ESUS. These predictors may help manage the ESUS population rather than the expensive ILR, especially in low-middle-income countries (LMICs).

Additionally, we assessed the prevalence of atrial cardiopathy in ESUS patients, which may contribute to thromboembolism even in the absence of AF.

## Methods

This prospective observational single-center study was conducted from June 2021 to January 2023. We included patients aged 18 years and older who were diagnosed with ischemic stroke and admitted to the stroke care unit, neuropsychiatry department, Tanta University Hospital, Egypt.

ESUS was diagnosed according to the consensus criteria defined by the Cryptogenic Stroke/ESUS International Working Group as the presence of non-lacunar ischaemic stroke, the absence of atherosclerosis causing ≥50% luminal stenosis, left ventricular ejection fraction (LVEF) ≥30%, and no identifiable cardioembolic source of embolism.^9^

Patients with a permanent pacemaker, an implanted cardiac defibrillator, recent cardiac surgery within 30 days of enrollment, and patients diagnosed with AF on a resting ECG done at enrollment or with a previous diagnosis of permanent AF or persistent AF were excluded.

All patients had neuroimaging and vascular investigations following a thorough clinical assessment and detailed history. Blood samples were collected to conduct routine laboratory tests. The cardiologic standard workup included resting ECG, seven days of inpatient cardiac Holter monitoring, and 2D transthoracic echocardiography.

Every patient received a standard 12-lead surface ECG, recorded at a speed of 25 mm/s and a 10 mm/mV voltage. A single observer who was blind to the patient group analyzed the ECGs at four times digital zoom.

The following P wave indices in ECG were defined and assessed as follows:

- PTFV1 was calculated as the product of the amplitude and the duration of the terminal downward deflection of the P-wave in lead V1.^10^ PTFV1 was only measured if the P-wave morphology in lead V1 had a negative or biphasic component and was excluded when there was a positive P-wave. Measurements were made on the admission paper ECG using digital calipers in mm and were then converted to μV and ms using the ECG calibration of 10 mm/mV and 25 mm/s.
- The P-wave durations were measured in lead V1 (PWDV1) and II (PWDII). It was defined as the time from the earliest commencement of P-wave activity in the leads to the latest P-wave activity in these leads. ^10^
- P-wave dispersion is defined as the difference between maximum and minimum P-wave durations measured at all ECG-derived leads.^11^

A 7-day in-hospital Holter monitoring was applied to all included patients to measure the prevalence of new-onset atrial fibrillation (NOAF). NOAF was defined as ECG (or Holter) evidence of irregular sustained rhythm with no evidence of discrete atrial activity in patients with a negative history of persistent or permanent AF.^12^

Each patient had 2D echocardiography utilizing a GE Vivid S5 machine with phased-array transducers (M5S) (General Electric, Horten, Norway) during the same hospital stay. According to the recommendations of the American Society of Echocardiography, standard measurements of several echocardiographic and Doppler parameters were taken with the determination of LVEF using Simpson’s biplane method, left atrial volume index (LAVI) calculated from apical four- and two-chamber views, and E/e^-^ ratio.^13^

Patients were divided into two groups according to the presence or absence of atrial cardiopathy: Group I, consisting of individuals diagnosed with atrial cardiopathy, and Group II, comprising those without atrial cardiopathy.

Atrial cardiopathy was defined by at least one of the following criteria: serum N-terminal pro-brain natriuretic peptide (NT-pro BNP) level greater than 250 Pg./ml; P-wave terminal force velocity in lead V1 (PTFV1) on ECG greater than 5000 µv.ms; or severe LAE on echocardiogram (LA size index 3≥ cm/m^2^).^6^

Subsequently, patients were divided into two subgroups based on the occurrence of NOAF: subgroup A, which had at least one episode of NOAF lasting longer than 30 seconds, and subgroup B, which had no episodes of AF.

Statistical analysis

- Continuous variables presented normal distribution (verified through the Kolmogorov-Smirnov test), being described by mean ± standard deviation and tested with the unpaired Student t-test.
- Categorical variables were presented as absolute and relative frequencies and analyzed using the χ^2^ test or Fisher’s exact test as appropriate.
- A multivariable logistic regression model adjusting for age, sex, hypertension, and diabetes mellitus was employed, and results were presented.
- Statistical analysis was done online using DATAtab: Online Statistics Calculator,^14^ where a p-value of <0.05 was considered statistically significant.

## Results

The initial identification of 82 consecutive patients with ESUS resulted in the exclusion of 7 patients because of technical issues in obtaining sufficient echocardiographic views (two cases), transfers to other hospitals before completing the Holter study for seven days (three cases), or in-hospital deaths (two cases). Thus, the study cohort was created by including the final 75 patients.

The included patients were then split into two groups based on whether or not they had atrial cardiopathy. Group I represented the population with atrial cardiopathy and included 40 patients, and Group II represented the population without atrial cardiopathy and included 35 patients. Subsequently, each group was subdivided into two subgroups, A and B, based on whether or not AF was present, respectively.

In-hospital NOAF episodes were recorded in 15 patients (representing 20% out of the total 75 ESUS), 7 patients in group I (17.5%), and 8 patients in group II (22.8%).

The average age of the included patients was 58 years old. Patients with atrial cardiopathy were significantly older than those with no atrial cardiopathy. The majority of patients were male. Hypertension was the most common comorbidity. People with diabetes were more likely to be in group II. There was no statistically significant difference between the two groups considering gender, hypertension, coronary artery disease, previous stroke/TIA, or Multiple vascular territories of infarction (Table 1).

**Table 1:** – Baseline clinical characteristics of the included population in the two groups

Variable	Group I: With atrial cardiopathy (n= 40)	Group II: Without atrial cardiopathy (n= 35)	p Value*
Age	60.5±6.39	55.14±3.57	<0.01
Male gender	25 (62.5%)	20 (57.1%)	0.637
Smoking	7 (17.1%)	8 (23.4%)	0.072
BMI (kg/m^2^)	27.6 ± 2.9	26.6 ± 3.1	0.126
Diabetes	6 (8%)	14 (18.67%)	0.015
Hyperlipidemia	17 (22.67%)	13 (17.33%)	0.637
Ischaemic heart disease	33 (44%)	27 (36%)	0.563
Heart failure	5 (11.9%)	5 (14.2%)	0.82
Previous TIA/Stroke	7 (9.33%)	8 (10.67%)	0.563
Multiple vascular territories infractions	7 (9.33%)	13 (7.33)	0.55

*A Chi^2^ test was performed. P value < 0.05 was considered statistically significant. BMI: body mass index.

Regarding echocardiographic information, there were no statistically significant differences when comparing LA diameter, LA diameter index, LA volume index, LA volume, LVEDVi, and LVMi, (Table 2).

**Table 2:** – Laboratory findings, echocardiographic data, and ECG characteristics in both groups

Variable	Group I: With Atrial Cardiopathy (N= 40)	Group II: Without Atrial Cardiopathy (N= 35)		p Value
Laboratory findings				
eGFR (mean ± Sd.)	82.43 ± 6.69	78.94±10.32		0.093
HBA1C	6.48±0.66	6.91±1.26		0.081
LDL-cholesterol	104.35±24.42	100.74±34.15		0.605
Echocardiographic findings				
LA diameter, mm	36.59 ±4.4	35.54±3.41		0.255
LA diameter index, mm/m^2^	21.45±2.53	20.85±1.99		0.265
LA volume, ml	53.68±10.38	53.66±9.09		0.994
LAVI, ml/m^2^	31.7±6.36	31.41±5.13		0.827
LVEDVi, ml/m^2^	58.5±2.72	59.13±3.42		0.375
LVMi, g/m^2^	98.4±2.99	98.03±4.53		0.677
**P wave indices in ECG**				
PTFV1, mV.ms	36.88±3.8	34.7±3.94		0.017
P wave duration, ms	113.7±0.83	113.43±0.94		0.182
P wave Dispersion	39.85±0.22	39.74±0.49		0.243

eGFR: estimated glomerular filtration rate; HBA1C: Glycated hemoglobin; LDL: low density lipoproteins; LA: left atrium; LVEDVi: LV end-diastolic volume index; LAVI: left atrial volume index; LVMi: left ventricular mass index; PTFV: P-wave terminal force in V1.

PTFV1 was statistically significantly higher in Group I than in Group II. However, the difference between the two groups as regards P wave duration and P-wave dispersion was not statistically significant.

Multivariable regression analysis was performed to determine independent predictors of atrial cardiopathy in ESUS patients. The results showed that hypertension and the E/e^-^ ratio were statistically significant (Table 3).

**Table 3:** – Multivariable regression analysis showing independent predictors of atrial cardiopathy in ESUS patients

Variable	p value	95% CI
Age	0.355	0.58 - 4.55
Smoking	0.467	0.28 - 1.8
Hypertension	0.001	2.01 - 17.88
HBA1C > 6.5 %	0.997	0 - Infinity
Dyslipidaemia	0.068	0.94 - 6.21
Prior MI	0.398	0.43 - 8.17
E/e-	0.02	1.2 – 8.19
LAVI, ml/m^2^	0.049	1 - 1.36
EF < 45%	0.998	0.57 - 1.5
P wave duration ( ms )	0.944	0.61 - 1.7
P wave Dispersion (ms)	0.799	0.32 - 4.31

HBA1C: Glycated hemoglobin; MI: myocardial infarction; LA: left atrium; LAVI: left atrial volume index; E/e-: Ratio of E to e- where E is Peak velocity of early diastolic transmitral flow and e- is Peak velocity of early diastolic mitral annular motion; EF: ejection fraction; PTFV1: P-wave terminal force in V1.

Regarding AF episodes, NOAF was recorded using seven days of Holter monitoring in 15 patients. The NOAF group showed a significantly higher LA volume index, an increased NT-pro BNP, and a higher PTFV1 compared to the patients without NOAF (Figure 1, Table 4).

**Figure 1:** – Box blot comparing A) LA volume index, ml/m2, B) NT-proBNP and C) PTFV1, mV.ms among patients with NOAF (+ NOAF) and patients without NOAF (- NOAF).

**Table 4:** – Comparing determinants of atrial cardiopathy (LA volume index, NT-proBNP, and PTFV1) between patients with NOAF and patients without NOAF

Variable	NOAF during 7 days of Holter monitoring n= 15	No NOAF during 7 days of Holter monitoring n= 60	p value*
LA volume index, ml/m^2^ (M ± SD)	38.8 ± 3.74	29.76 ± 4.69	<0.001
NT-proBNP pg/ml (M ± SD)	249.27 ± 66.52	326.53 ± 46.05	<0.001
PTFV1,mV.ms (M ± SD)	42.53 ± 0.55	34.02 ± 2.29	<0.001

NOAF: New-onset atrial fibrillation; NT-proBNP: N-terminal pro-atrial natriuretic peptide; PTFV1: P-wave terminal force in V1. * T-test was used. P value <0.05 was considered significant.

In order to identify the independent determinants of AF in ESUS patients with atrial cardiopathy, a multivariable regression analysis was performed. We found that PTFV1 > 5000 mV.ms, LA volume index > 34 ml/m^2^, and ejection fraction <45% were significant independent predictors for AF (Table 5).

**Table 5:** – Multivariable regression analysis showing independent predictors of atrial fibrillation in ESUS patients with atrial cardiopathy

	p value	95% CI
Age > 65	0.175	0.54 - 25.93
Smoking	0.4	0.07 - 3.01
Hypertension	0.186	0.45 - 4.34
HBA1C > 6.5 %	0.877	0.08 - 8.77
Dyslipidaemia	0.173	0.43 - 41.8
Prior Myocardial Infarction	0.137	0.65 - 40.95
NT-pro BNP>250 pg/ml	0.087	0.61 - 59.45
E/e- > 12	0.038	0 - Infinity
LA size index 3 ≥ cm/m^2^	0.062	0 - Infinity
LAVI > 34 ml/m^2^	<0.001	0 - Infinity
EF < 45%	0.001	0.43 - 0.99
PTFV1 > 5000 mV.ms	0.001	1 - 1.02
P wave duration ( ms )	0.014	0.11 - 0.83
P wave Dispersion (ms)	0.433	0.11 - 121.32

HBA1C: Glycated hemoglobin; NT-proBNP: N-terminal pro-atrial natriuretic peptide; E/e-: Ratio of E to E’ where Peak velocity of early diastolic transmitral flow and e- is Peak velocity of early diastolic mitral annular motion as determined by pulsed wave Doppler; LA: left atrium; LAVI: left atrial volume index; EF: ejection fraction; PTFV1: P-wave terminal force in V1.

## Discussion

In 2014, the concept of embolic stroke of undetermined source (ESUS) was developed and published. Approximately 20% of all ischaemic strokes are caused by embolic stroke of an undetermined source (ESUS), a subtype of cryptogenic ischaemic stroke.^15,16^

AF is significantly correlated with ischemic strokes, contributing to global morbidity and mortality.^1^ The purpose of this study was to find the potential predictors of paroxysmal AF in patients who were in sinus rhythm at the time of stroke diagnosis. This will aid in the formulation of a preventive strategy aiming at mitigating the occurrence of embolic strokes.

NOAF episodes were recorded in 20% of the total 75 ESUS patients. This rate of NOAF identification is similar to that found by Vera et al.^17^ who detected AF in 24% of patients who underwent 15 days of wearable Holter-ECG monitoring. Moreover, this occurrence has a resemblance to the study conducted by Carrazco et al.^18^, who found that 31% of 101 patients with cryptogenic stroke had AF while being monitored by ILR.

In other studies, the rate of identification of NOAF using inpatient Holter monitoring for 24 hours, 48 hours, and 7 days is 4.6%, 6.4%, and 12.5%, respectively, whereas the rate of detection using an implantable loop recorder for 6 months is 9%.^19,20^

In our study, 53.3% of ESUS patients had atrial cardiopathy. This proportion is comparatively higher than the findings reported by Ning et al.^21^ and Jalini et al.^22^, who reported prevalence rates of 42.8% and 26.6%, respectively. Nevertheless, the diagnostic criteria for atrial cardiopathy proposed by Shirin Jalini et al. were limited to the presence of a p-wave terminal force in V1 greater than 5,000 µV.ms or severe LAE. Notably, these criteria did not incorporate the inclusion of high NT-proBNP levels over 250 pg/ml as a diagnostic criterion.

Regarding PTFV1, our result matches that of Hancock et al.^23^, who found that abnormally increased PTFV1 is a biomarker of left atrial dysfunction and is associated with pathophysiological changes such as hypertrophy and elevated filling pressures. Evidence from observational cohorts suggests that elevated PTFV1 is related to the risk of ischemic stroke.^24^

As regards large LAVI > 34 ml/m^2^, the association could be explained by the fact that LAE carries a thrombogenic potential likely by promoting stasis, endothelial injury, and thrombus formation. This is in accordance with Jordan et al.^25^, who revealed that the LAVI to the subject’s body surface area is independently associated with the development of AF in ESUS patients. Additionally, our results agree with Yaghi et al.^6^, who explained the association between LAE and recurrent embolic stroke subtype.

Elevated NT-proBNP is a measure of cardiac dysfunction, cardiac stretch, and volume overload and is a predictor of incident AF.^21^ In our study, elevated NT-pro BNP > 250 pg/mL was found in 35 patients; of them, only 7 patients had AF episodes during their monitoring. These results match those of Chen et al.^26^ who found an association between elevated NT-proBNP and ischemic stroke, particularly of the embolic subtype.

In contrast to Handke et al.^27^ who showed that AF is likely to be more prevalent among older (>60 years) patients with ESUS, we did not identify age as a predictor for AF. This could be attributed to our small sample size, which limited our results.

A group of ESUS patients may benefit from OAC, as demonstrated by Diener et al.^15^ particularly those with atrial cardiopathy. Additionally, Merkler et al.^28^ found that rivaroxaban was superior to aspirin in reducing the risk of recurrent stroke or systemic embolism among NAVIGATE ESUS participants with LV dysfunction. The Canadian guidelines recommend that older patients with non-lacunar ESUS and suspected AF undergo extensive AF screening to identify patients who would benefit from OAC if a clear diagnosis is found.^12^

As long as the RE-SPECT ESUS^29^ and NAVIGATE ESUS^30^ studies have raised concerns about the safety of anticoagulation in patients with ESUS, we recall the importance of assessing individual bleeding risk using the HAS-BLED score.^31^ Renal function and complete blood count should be measured before starting OAC and at regular intervals after that while the prescribed OAC is dosed appropriately.

### Study limitation

1. The conducted study was a single-center investigation.
2. The study involved a relatively limited number of cases.
3. A single observer conducted the ECG analysis.

## Conclusion

In ESUS patients, atrial cardiopathy was prevalent. Hypertension and an E/e^-^ ratio greater than 12 were independent predictors for it. Multivariable regression analysis identified PTFV1 > 5000 mV.ms, LA volume index > 34 ml/m^2^, and ejection fraction < 45% as independent predictors for NOAF.
